# Understanding paratyphoid infection: study protocol for the development of a human model of *Salmonella enterica* serovar Paratyphi A challenge in healthy adult volunteers

**DOI:** 10.1136/bmjopen-2014-007481

**Published:** 2015-06-16

**Authors:** David McCullagh, Hazel C Dobinson, Thomas Darton, Danielle Campbell, Claire Jones, Matthew Snape, Zoe Stevens, Emma Plested, Merryn Voysey, Simon Kerridge, Laura B Martin, Brian Angus, Andrew J Pollard

**Affiliations:** 1 Oxford Vaccine Group, Department of Paediatrics, University of Oxford, and the NIHR Oxford Biomedical Research Centre, Oxford, Oxfordshire, UK; 2 Primary Care Clinical Trials Unit, Nuffield Department of Primary Care Health Sciences, University of Oxford, Oxford, Oxfordshire, UK; 3 Novartis Vaccines Institute for Global Health, Siena, Italy; 4 Nuffield Department of Medicine, University of Oxford, Oxford, Oxfordshire, UK

**Keywords:** MICROBIOLOGY, BACTERIOLOGY, IMMUNOLOGY, INFECTIOUS DISEASES

## Abstract

**Introduction:**

This study will develop the first human challenge model of paratyphoid infection which may then be taken forward to evaluate paratyphoid vaccine candidates. *Salmonella* Paratyphi A is believed to cause a quarter of the estimated 20 million cases of enteric fever annually. Epidemiological evidence also suggests that an increasing proportion of the enteric fever burden is attributable to *S*. Paratyphi infection meriting further attention and interest in vaccine development. Assessment of paratyphoid vaccine efficacy in preclinical studies is complicated by the lack of a small animal model and the human-restricted nature of the infection. The use of experimental human infection in healthy volunteers provides an opportunity to address these problems in a cost-effective manner.

**Methods and analysis:**

Volunteers will ingest virulent *S*. Paratyphi A bacteria (NVGH308 strain) with a bicarbonate buffer solution to establish the infectious dose resulting in an ‘attack rate’ of 60–75%. Using an a priori decision-making algorithm, the challenge dose will be escalated or de-escalated to achieve the target attack rate, with the aim of reaching the study end point while exposing as few individuals as possible to infection. The attack rate will be determined by the proportion of paratyphoid infection in groups of 20 healthy adult volunteers, with infection being defined by one or more positive blood cultures (microbiological end point) and/or fever, defined as an oral temperature exceeding 38°C sustained for at least 12 h (clinical end point); 20–80 participants will be required. Challenge participants will start a 2-week course of an oral antibiotic on diagnosis of infection, or after 14 days follow-up.

**Ethics and dissemination:**

The strict eligibility criterion aims to minimise risk to participants and their close contacts. Ethical approval has been obtained. The results will be disseminated in a peer-reviewed journal and presented at international congresses.

**Trial registration number:**

NCT02100397.

**Strengths and limitations of this study**

- The development of a *Salmonella* Paratyphi human challenge model should expedite the development and evaluation of potential paratyphoid vaccine candidates, in particular by allowing the direct measurement of vaccine protective efficacy in a safe, reproducible host-relevant model.
- Studying the longitudinal physiological and immunological responses to infection will provide insight into this increasingly prevalent but poorly understood infection.
- The modest number of participants in challenge studies can make the model sensitive to individual variation.
- The response to challenge may not reflect the target population who may have protection due to repeated natural exposure.
- The immunobiological response to *S*. Paratyphi A exposure may differ between strains.

## Introduction

Enteric fever is the term used to describe systemic illness caused by infection with *Salmonella enterica* serovars Typhi and Paratyphi A and C. Enteric fever is a leading cause of morbidity worldwide, particularly among young, school-aged children in resource-limited settings and increasingly among travellers to those areas.^1^ ^2^

While infection with *S*. Typhi accounts for the majority of enteric fever cases, the proportion of disease caused by *S*. Paratyphi A is increasing, particularly in the highly endemic regions of Southeast Asia and the Indian subcontinent.^3^ As a human-restricted pathogen, the targeted vaccination of those groups at high risk is likely to have a substantial impact on disease incidence.^4^

In view of the high burden of enteric fever and increasing antibiotic resistance, the WHO has stated that countries with high-risk groups and populations ‘should consider the programmatic use of typhoid vaccines for controlling endemic disease’.^4^ Despite this recommendation, there has been a reluctance to introduce programmes with the available licensed typhoid vaccines, Vi polysaccharide (ViPS) and Ty21a, as both have limited efficacy, offer minimal cross-protection against paratyphoid infection, and cannot be given to children less than 2 years of age. This has focused development on firstly conjugate *S*. Typhi and Paratyphi A vaccines, which can be administered to infants and expected to provide long-lasting immune memory, as well as attenuated oral vaccines.^5^ ^6^ A number of promising candidate vaccines are in early phase testing.^5–8^

The aim of this study is to develop a safe, reliable human paratyphoid challenge model in which to validate vaccines and develop novel diagnostics. Human challenge studies with *S*. Typhi (Quailes strain) have been performed historically and recently using an adapted model.^9–11^ To the best of our knowledge, human challenge with virulent *S*. Paratyphi has not been performed previously, so this study will provide a unique opportunity to study human paratyphoid infection. The study is designed to determine the dose of *S*. Paratyphi A (challenge strain NVGH308) required to produce an infection attack rate of 60–75% in healthy adult volunteers who have not previously been exposed to typhoidal salmonella and are therefore immunologically naive to the challenge agent. With sufficient evidence of paratyphoid infection at a target dose, this model can then be taken forward to evaluate paratyphoid vaccine candidates.

## Methods/design

**Figure 1:**
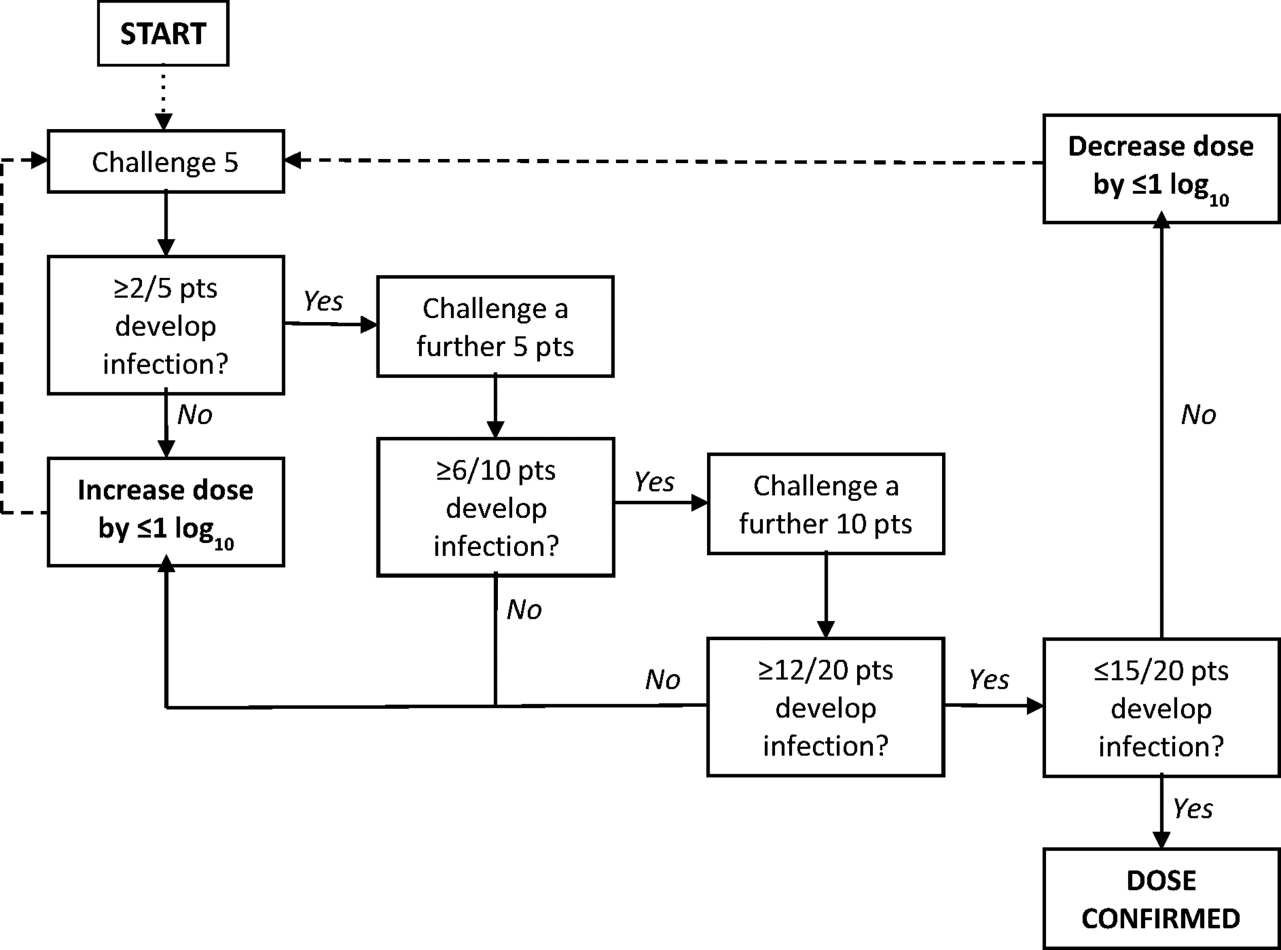
Decision-making algorithm for *Salmonella* Paratyphi dose escalation/de-escalation, starting at 1–5×10^3^, to reach the primary end point (pts, patients).

This is a descriptive, dose-level escalation human infection study using the *S*. Paratyphi A challenge of ambulatory, outpatient healthy community adult volunteers. For safety, the study will start with the challenge of one individual at an initial dose of 1–5×10^3^ colony forming units (CFU). Consecutive groups of 5 or 10 participants will then be challenged a minimum of 2 weeks apart following a decision-making algorithm for dose escalation/de-escalation (see figure 1). This algorithm was used for the typhoid challenge model developed by the University of Oxford in 2009.^9^

### Regulation and governance

In the UK, investigational products, such as unlicensed medications and vaccines, are regulated by the Medicines for Human Use (Clinical Trials) Regulations 2004 which implement the European Clinical Trials Directive (2001/20/EC).^12^ However, most microbial challenge studies fall outside the remit of the European Clinical Trials Directive (ECTD) and are instead judged according to common law and best practice.^13^ The typhoid and paratyphoid challenge models, using a fully characterised and non-genetically modified strain, falls under this category. To achieve best practice, challenge protocols undergo independent and rigorous peer review to assess the scientific quality and appropriateness of the study methodology to answer the key objectives. This includes conforming to Good Clinical Practice (GCP) guidelines with support from a research ethics committee experienced in these studies.^14^ As such, local or regional ethics committees, independent of the researchers and sponsors, are fundamental for research governance of microbial challenge studies.

### Challenge strain

The original *S*. Paratyphi A strain NVGH308 was isolated in 2006 from a patient with acute paratyphoid fever as part of a study performed by the Oxford University Clinical Research Unit at Patan Hospital, Kathmandu, Nepal. It has been manufactured to Good Manufacturing Practice (GMP) standard and is supplied for study purposes by Novartis Vaccines for Global Health, Italy. Manufacturing to GMP standard, while not a regulatory requirement in the UK, fulfils best practice.^13^

A parent seed lot, S888P5SP01, was established in March 2010 after serial colony selections on Luria Broth PTK agar plates and stored in the Novartis Vaccines and Diagnostics bacterial seed bank (Siena, Italy). This seed lot underwent five sequential passages as part of the cell line cleaning process before being used to establish the GMP Master Cell Bank, SA-13-002, of the NVGH308 strain. GenIbet BioPharmaceuticals (Oeiras, Portugal) produced three dose levels of the challenge agent under GMP conditions. Prepared vials containing the challenge agent were stored at −80°C±5°C and transferred via an accredited courier to the Oxford Vaccine Group Laboratory in 2013.

Batch testing of the cell bank confirms O:1 and O:2 antigen positivity, batch purity and stable viable bacterial count. Ongoing stability testing of the seed lot has been performed by Novartis Vaccines and Diagnostics. The NVGH308 strain is fully characterised with known susceptibility to a number of antimicrobial options.

### Study objectives

The primary objective is to determine the dose (in CFU) of *S*. Paratyphi A, challenge strain NVGH308, needed to produce a 60–75% attack rate when ingested with sodium bicarbonate solution by healthy adult volunteers. The secondary objectives will describe the interaction between bacteria and human host at baseline, through inoculation and symptomatic infection (or asymptomatic immune protection) to recovery and long-term follow-up (see table 1).

**Table 1:** Study objectives and outcomes

	Objective(s)	Outcome/end point(s)
Primary	To determine the dose (in colony forming units) of *Salmonella* Paratyphi A, challenge strain NVGH308, needed to produce a 60–75% attack rate when ingested with sodium bicarbonate solution in healthy adult volunteers	Clinically or microbiologically proven paratyphoid infection following oral challenge with *S*. Paratyphi A, strain NVGH308, delivered with sodium bicarbonate solution
Secondary	(1) To describe the human physiological response to *S*. Paratyphi A challenge, and in those developing or not developing infection	Description of the clinical course after challenge using, for example, participant symptom profiles, temperature measurements and other recorded clinical and laboratory observations
	(2) To evaluate the sensitivity of the predefined criteria for paratyphoid infection, using subsequent clinical, microbiological and laboratory outcomes	Determination of the challenge dose/kg (dose/surface area) actually ingested by those developing and those not developing paratyphoid infection at each dose level. Analysis of the attack rate using alternative criteria including, for example, passive field surveillance definitions, alternative temperature thresholds and adjunctive microbiological and laboratory diagnostic assays
	(3) To describe the characteristics of bacterial dynamics after challenge, including onset and duration of bacteraemia, bacterial burden at diagnosis and stool shedding	Microbiological assays to detect and characterise *S*. Paratyphi after challenge in blood, stool and urine
	(4) To describe the human immune response to challenge, including the innate, humoral, cell-mediated and mucosal responses	Immunological laboratory assays to measure innate, humoral, cell-mediated and mucosal responses to challenge
	(5) To determine genetic features affecting host–pathogen responses, alteration of those responses through epigenetic changes, control of gene expression and post-translational modifications	Laboratory and high-throughput assays to measure genetic factors affecting susceptibility, gene expression and protein translation
	(6) To discover, develop and evaluate novel diagnostic methods for *S*. Paratyphi A infection	Exploratory analysis of blood, faeces, saliva and urine samples using experimental assays and diagnostics
	(7) To explore the factors, influences and motivation affecting volunteers’ decision to participate in human challenge studies and their experiences of the study process	Participant responses using questionnaires during the course of the study

Feedback and recommendations from Patient and Public Involvement (PPI), in addition to participant questionnaires from previous studies, have been incorporated into this study. This beneficial process will continue with an anonymous questionnaire to be completed by participants 28 days after challenge.

The questionnaire will be closely based on the one used by the Oxford Vaccine Group (OVG) in previous typhoid studies to explore participants’ study experience. This includes motivations, attitudes and factors influencing participation in human challenge research, as well as their experience of study procedures such as ingesting the challenge agent.

### Study setting

The study will be conducted at the Centre for Clinical Vaccinology and Tropical Medicine, Oxford, UK, which is a fully equipped vaccine research site with available clinical inpatient facilities and a Category III level laboratory on-site. The UK is non-endemic for enteric fever with the majority of cases travel related; the rate of paratyphoid fever notification in the Thames Valley region, which includes Oxfordshire, is 0.5/100 000.^15^

### Recruitment

Several strategies may be employed in order to recruit the required cohort of volunteers. These include:

- Study invitation letters with reply slips, sent out by the National Health Applications and Infrastructure Services who hold the central National Health Services (NHS) patient database (Open Exeter),
- Website and poster advertising,
- Direct mail-out using the Electoral register,
- Email communication to local tertiary education facilities.

The Oxford Vaccine Centre also manages a secure database for healthy volunteers who have expressed an interest in being contacted about potential studies. Potential participants will be invited for a screening and consent visit, where a member of the clinical team will assess their eligibility.

Participants will be offered reimbursement for their time, travel and inconvenience. The amount, frequency and method of payment will be described in the study information booklet. The payment schedule will hopefully encourage attendance of follow-up appointments.

### Eligibility criteria

Male or female participants, aged 18–60 years inclusive, who are in good health (as determined by a study doctor, medical investigation and agreement by their general practitioner) and able to provide written informed consent, will be eligible for inclusion in this study. Additional inclusion and exclusion criteria will be applied to ensure that participants are appropriate for the study (see online supplementary 1). This includes a participant's ability to attend daily appointments for 2 weeks after challenge with *S*. Paratyphi A. These strict criteria aim to minimise the risk of severe or complicated disease in participants and reduce the potential risk of transmission to close contacts.

### Interventions

A participant will be considered enrolled in the study on their day of challenge.

The method used for preparation of the challenge inocula is based on the one used for the recent typhoid challenge model.^9^ Participants will fast for 90 min before ingesting 120 mL of sodium bicarbonate (2.1 g NaHCO_3_) to neutralise stomach acid. This is followed 60 s later by the challenge inocula which will be freshly prepared prior to each challenge by defrosting and suspending the required bacterial dose in 30 mL of sodium bicarbonate (0.53 g NaHCO_3_).

**Figure 2:**
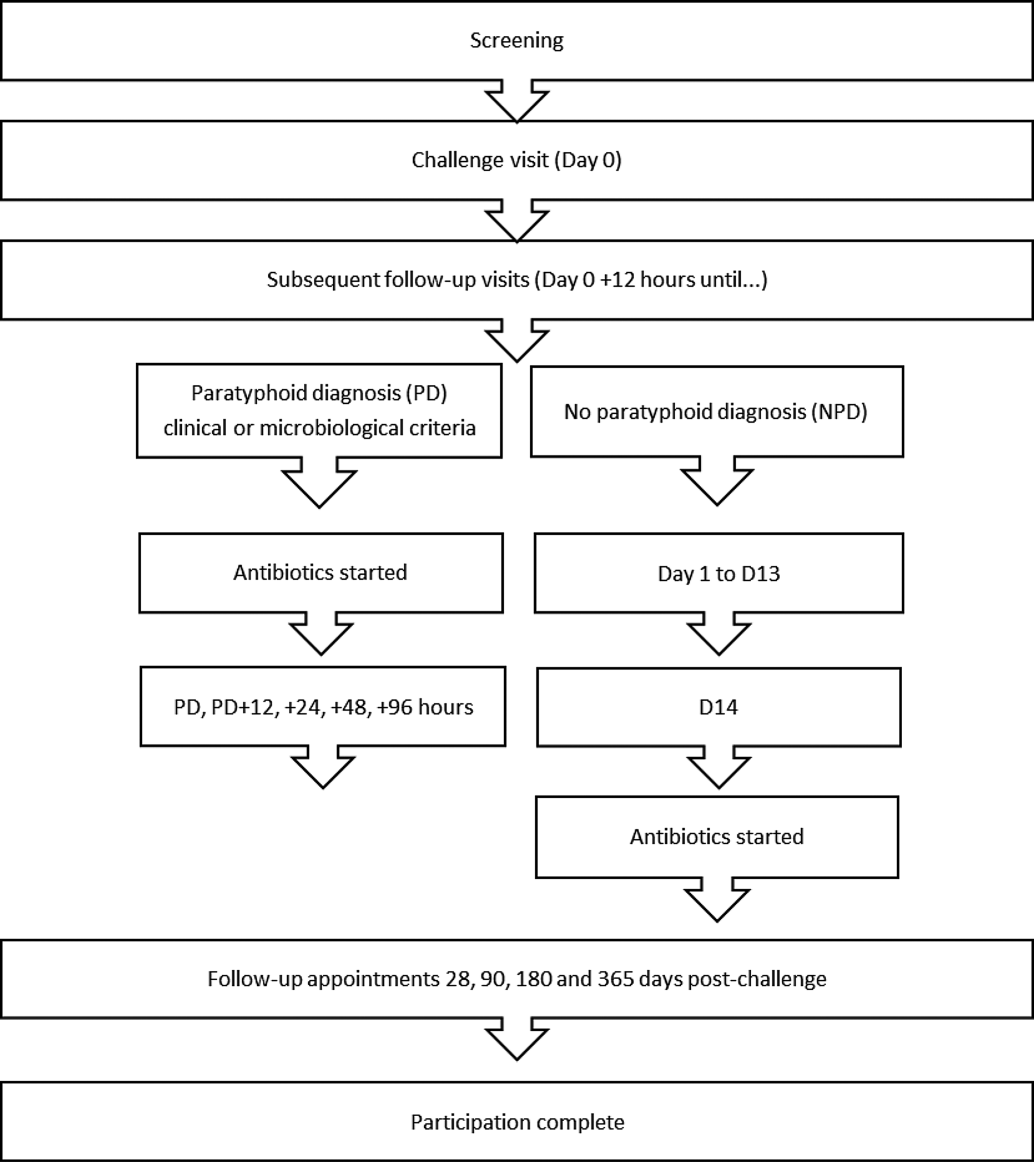
Participant journey through the study.

After *S*. Paratyphi A ingestion, participants will be seen daily for 14 days with blood, stool, saliva and urine samples taken at set time points (see online supplementary 2). Monitoring for derangement of liver, renal, blood count parameters and inflammatory markers will be performed. Participants will also complete twice-daily temperature readings and record any symptoms experienced for 21 days; these data will be collected by electronic case report forms (eCRFs) and diary cards. Subsequent follow-up appointments will be 28, 90, 180 and 365 days after challenge (figure 2).

Paratyphoid infection will be diagnosed after challenge if one of the following applies:

- A positive blood culture for *S*. Paratyphi from 72 h after challenge,
- A positive blood culture for *S*. Paratyphi within 72 h, with one or more signs/symptoms of paratyphoid infection,
- Persistent positive blood cultures for *S*. Paratyphi within 72 h,
- An oral temperature>38°C persisting for 12 h.

The earliest microbiological indication that a participant has an *S*. Paratyphi A bacteraemia will be the identification of a Gram-negative bacillus (GNB) from a positive blood culture. As formal identification of the organism may take a minimum of a further 24 h, participants in whom a GNB is identified will be defined as having paratyphoid fever.

Ciprofloxacin 500 mg twice daily for 14 days will start on diagnosis of paratyphoid infection or at day 14 after challenge. The rationale for using fluoroquinolones as a first-line agents is based on recommendations for treatment of enteric fever and prevention (and treatment) of chronic gallbladder carriage as therapeutic levels are reached in bile and gallbladder.^16^ ^17^ All positive blood culture isolates will have full susceptibility profiling performed using an antibiotic disc method and Etest to measure the ciprofloxacin minimum inhibitory concentration (MIC). Alternative antibiotics used as second-line therapy (in the event of adverse reactions or side effects) include azithromycin and trimethoprim/sulfamethoxazole.

If the participant's symptoms fail to resolve after antibiotic administration, if they are unable to tolerate oral antibiotics, become dehydrated, or if unanticipated concerns regarding home circumstances emerge, inpatient admission to an infectious diseases unit will be considered. Patient care at this stage would be delegated to the hospital clinical team, which could include the provision of intravenous fluids, antibiotics and antiemetics.

### Safety

*Participant safety during the study*: Participants will be monitored closely with daily clinical review and completion of symptom diary cards. All adverse events will be recorded on CRFs, with serious events notified to the Data Safety Monitoring Committee (DSMC) within 24 h of the investigator becoming aware of the event. Adverse events of special interest (AESI) will also be reported to the DSMC in the same manner and include:

- Complications of paratyphoid fever such as perforation or haemorrhage which occurs almost exclusively in patients who are untreated for an extended period,^18^
- Failure to clinically or bacteriologically cure a participant of acute paratyphoid infection within 14 days of antibiotic therapy,
- Relapse or progression to carrier state, the latter defined as a person who is still excreting *S*. Paratyphi A after two courses of appropriate antibiotic therapy,^19^
- Transmission of *S*. Paratyphi to non-study participants.

The DSMC for this study will be composed of an independent and trial-experienced group of infectious disease and public health clinicians and a statistician. Safety data collated from eCRFs and eDiaries, which include blood parameters, vital signs and symptom recordings, will be reviewed by the DSMC after the first participant has been challenged and at each dose escalation. Approval from the DSMC will be required prior to any subsequent dose alterations. This role and function of the DSMC will be described in a Charter, agreed prior to the start of recruitment.

*Long-term safety of participants*: The risk of chronic carriage with *S*. Paratyphi A is minimised by treatment for 2 weeks with an effective antibiotic and excluding participants with gallbladder disease.^20^ In addition, stool samples for culture will be obtained 2 weeks after completion of the antibiotic course and then weekly until two successive samples are negative. If samples remain positive for *S*. Paratyphi A 4 weeks after completion of antibiotics, then the participant will be referred to a NHS infectious diseases consultant for further management.

*Safety of non-study participants*: The risk of secondary transmission to close contacts is unlikely in view of the low infectivity of *S*. Paratyphi A and the level of hygiene and sanitation in the UK.^21^ Consent will not be obtained from close household contacts, but participants will be required to provide them with a written study summary detailing measures to reduce the risk of infection and offering screening for paratyphoid infection. Even in the absence of transmission precautions, the rate of secondary cases is exceptionally low within the UK.^22^

The participants will consent to the clinical study team informing the local Health Protection Unit of their involvement in the study. The Unit will be notified of their challenge date and when stool clearance has been completed. Any breach in enteric hygiene precautions that result in another individual coming into contact with infectious material will be reported, with potential cases of transmission to be confirmed by sequence comparison to an isolate of the challenge strain stored at a Public Health England microbiology reference laboratory (Colindale, London).

### Sample size

The selected sample size balances the need for a statistically reproducible attack rate while minimising the number of individuals exposed to paratyphoid infection. To meet the primary objective of a clinically reproducible attack rate of 60–75%, this careful, dose (de-)escalation protocol will be followed.^9^ The maximum number of participants required will be 80, with the minimum 20 if the starting dose (1–5×10^3^ CFU) satisfies our criteria. This is based on the probability that the criteria are satisfied according to the true attack rate.

If the attack rate in the first group of 20 participants is greater than 75%, a lower dose will be decided based on the prior attack rate combined with laboratory and clinical findings. De-escalation to a dose lower than 1–5×10^3^ CFU will also be considered if the target attack rate is reached and the chief investigator, with agreement from the DSMC, decides that a lower dose may achieve a similar attack rate.

### Statistical analysis

The analysis of the primary end point will be descriptive only. The percentage of participants who meet the criteria for diagnosis of paratyphoid will be calculated with a 95% Clopper-Pearson Exact CI. Those individuals who withdraw or are treated prior to day 14 without prior diagnosis of paratyphoid would be excluded from this analysis. A secondary analysis of the primary end point will be conducted using the Kaplan-Meier method which will include all participants.

Time-to-event analyses of individual components of the primary outcome (eg, positive blood culture for *S*. Paratyphi, oral temperature>38.0°C, etc) will be conducted using the Kaplan-Meier method and will include all participants. Participants not meeting the criteria for an individual component of the primary end point will be censored in the analysis at the time of diagnosis or at day 14 for those undiagnosed.

## Discussion

This will be the first *S*. Paratyphi A human challenge model developed. It is presumed that this study will be similar to the experience of recent typhoid challenge studies based on the literature iterating the similar clinical presentation between typhoid and paratyphoid fever.^9^ ^23^ ^24^ Clinical knowledge, however, of the NVGH308 paratyphoid A strain is limited to details from the original patient, in contrast to the *S*. Typhi Quailes strain where data from 762 challenged participants from the 1960s informed the re-establishment of a typhoid challenge model in 2009.^25^ While the lowest infective inoculum of *S*. Paratyphi A is unknown, it is believed to be similar to or higher than *S*. Typhi.^26^ ^27^ As such, we will use the same starting dose of 10^3^ CFU that was used in the 2009 typhoid challenge model. This dose was the highest dose of *S*. Typhi that did not cause clinical infection in the historical typhoid challenge studies.^28^ When coadministered with the sodium bicarbonate solution, 10^3^ CFU *S*. Typhi (Quailes strain) gave an attack rate of 55%, necessitating a dose escalation to reach the target attack rate of 60–75%.^9^ We anticipate that the same will occur with the *S*. Paratyphi A challenge.

The outpatient management of participants challenged with typhoid is safe and achievable; this has been key in re-establishing the challenge model due to the prohibitive cost of inpatient care.^9^ Participant satisfaction with this model, plus the monetary reimbursement for their time, travel expenses, blood draws and potential days off work, is high.^29^

The modest number of participants in challenge studies can make the model sensitive to individual variation. Selecting an antigen-naive cohort limits this variation, but the response may not reflect the target population who may have protection due to repeated natural exposure, with consequent overestimation of the potential efficacy of a candidate vaccine.^30^ Conversely, the challenge dose required to achieve a sufficiently high attack rate within a manageable 2-week period is likely to be higher than was encountered in the field. This may overwhelm candidate vaccines with erroneously discouraging protective efficacy, as was seen in the Maryland typhoid studies.^31^

The NVGH308 strain, while originally a clinical isolate, may not be representative of the current circulating strains in endemic settings. *S*. Paratyphi A, however, is a clonal monomorphic pathogen containing limited genomic variation,^32^ ^33^ making it likely that the pathogenicity and immune response to the NVGH308 *S*. Paratyphi A challenge will translate to wild-type strains and that future vaccines will provide cross-strain protection.

Current promising candidate *S*. Paratyphi vaccines are based on whole cell live-attenuated strains or subunit approaches that conjugate O polysaccharide (O:2) to a range of protein carriers. This O:2 polysaccharide antigen of *S*. Paratyphi A is known to play a role in protection and virulence.^34^ A phase II trial is underway of O:2 conjugated to tetanus toxoid (O:2-TT), conducted after initial trials showed that it was safe and immunogenic.^5^ A second conjugate vaccine moving into clinical testing is O:2 conjugated to CRM_197_ (O:2-CRM_197_), which demonstrated immunogenicity in preclinical studies with strong bactericidal activity against *S*. Paratyphi A when developed alone or in combination with Vi-CRM_197._^7^ A live-attenuated oral vaccine candidate (Centre for Vaccine Development (CVD) 1901, University of Maryland) has also been shown to be well tolerated and immunogenic in phase I trials and further phase I studies are ongoing.^8^

Although promising vaccines are in development, it is a long and costly process for any vaccine to get to licensure. The lack of a reliable correlate of protection and the poorly understood immunobiology of typhoid and paratyphoid infection adds to the difficulties in enteric fever vaccine development. Equally, highly sensitive and specific diagnostic tests for use in endemic settings are needed, but their development and, particularly, validation has been hindered by the lack of a patient cohort immunologically naive to typhoidal salmonella. Advancing knowledge on the microbiological and human–host response to exposure is necessary to inform transmission and impact modelling for vaccine roll-out, key for targeted vaccination programmes of high-risk population groups.

The development of an *S*. Paratyphi A human challenge model could help overcome some of these limitations. As trials for paratyphoid and bivalent vaccine candidates are approaching Phase I/II stages, a paratyphoid challenge model could provide a crucial intermediate step in progressing efficacious vaccine candidates into more expansive field trials in endemic settings. Ideally, this will be translated into rapid, cost-effective diagnostics, contributing to the disease surveillance necessary for vaccination programmes. Future measures to control enteric fever are expected to combine an effective bivalent vaccine against both serovars with public health measures that improve sanitation and access to clean water.
